# Personalized Pain Management in Orthodontic Treatment: A Narrative Review of Biological, Psychological, and Clinical Determinants

**DOI:** 10.7759/cureus.113272

**Published:** 2026-07-24

**Authors:** Neslihan Karaoglan

**Affiliations:** 1 Orthodontics, Health Sciences University Sultan Abdulhamid II Training and Research Hospital, Istanbul, TUR

**Keywords:** clear aligners, dental anxiety, fixed appliances, orthodontic pain, pain catastrophizing, pain perception, personalized pain management, photobiomodulation

## Abstract

Orthodontic pain is a frequent and clinically relevant experience during treatment, particularly after separator placement, bonding, archwire insertion, appliance activation, and elastic use. Although pain is often transient, it may negatively affect comfort, cooperation, diet, oral hygiene, satisfaction, and adherence. Orthodontic pain is not determined solely by mechanical tooth movement; it reflects a complex interaction between inflammatory tissue responses, individual pain sensitivity, psychological status, previous dental experiences, appliance type, and procedure-related factors. This narrative review summarizes the biological, psychological, and clinical determinants of orthodontic pain and discusses current pharmacological and nonpharmacological pain management strategies. Based on the available evidence, a proposed conceptual framework for personalized orthodontic pain management is presented. This framework stratifies patients into low-, moderate-, and high-risk profiles according to biological sensitivity, anxiety, pain expectation, treatment stage, and clinical complexity. Low-risk patients may require reassurance and basic instructions, whereas moderate- and high-risk patients may benefit from closer follow-up, individualized education, short-term analgesic planning, and selected adjunctive strategies. This framework should be interpreted as a conceptual model rather than a validated clinical prediction tool. Future prospective studies are needed to validate practical risk assessment instruments and individualized pain control protocols in orthodontic patients.

## Introduction and background

Orthodontic pain is one of the most frequently reported adverse experiences during orthodontic treatment and can influence patient attitudes, cooperation, satisfaction, diet, oral hygiene, sleep, daily activities, and willingness to continue treatment [1-3]. It is commonly reported after separator placement, bonding, initial archwire insertion, activation appointments, intermaxillary elastic use, and appliance removal. Classic clinical studies and more recent fixed-appliance trials indicate that pain often begins within the first few hours, reaches its highest level during the first day or the first 24-48 hours, and gradually decreases over the following days [4-7]. Recent systematic evidence in adult fixed-appliance patients also confirms that pain and discomfort during early treatment can be accompanied by a decline in oral health-related quality of life (OHRQoL) [8].

The biological basis of this pain is related to the response of periodontal and alveolar tissues to orthodontic force. Mechanical loading produces areas of compression and tension in the periodontal ligament, leading to vascular changes, transient ischemia, inflammatory mediator release, and sensitization of peripheral nociceptors [2,9]. Pain signals from periodontal and oral tissues are then transmitted through trigeminal afferent pathways to central nervous system structures involved in sensory-discriminative and affective-emotional pain processing. However, the perceived intensity of orthodontic pain is not explained by tissue response alone. Anxiety, anticipation of pain, previous dental experiences, catastrophizing, coping confidence, and attention to symptoms can modulate pain perception and the functional burden experienced by the patient [10-13].

Procedure-related factors are also important. Bonding and initial archwire placement may represent the patient’s first experience of fixed appliance discomfort, separators can impair chewing before molar banding, elastics may interfere with adherence, and debracketing may generate pain or anxiety at the end of treatment [7,14,15]. Separator-related interventions have recently been synthesized in a systematic review and meta-analysis, which highlighted both the clinical relevance of separation pain and the heterogeneity of available pharmacological and nonpharmacological approaches [16]. Appliance type may further modify the pain experience; fixed appliances, lingual appliances, and clear aligners can produce different pain trajectories because of differences in force delivery, mucosal irritation, removability, hygiene demands, and patient behavior [17-19].

These observations support a broader and more individualized approach to orthodontic pain management. Traditional pain control often focuses on analgesic use after pain occurs; however, a risk-based model begins before the procedure by identifying patients likely to experience clinically important discomfort. The objective of this narrative review is to summarize biological, psychological, and clinical determinants of orthodontic pain, appraise available pain management strategies, and propose a conceptual management pathway that links patient-level risk features with procedure-specific counseling, analgesic planning, adjunctive options, and follow-up intensity. The novelty of this framework is that it translates the general biopsychosocial understanding of pain into a practical orthodontic pathway organized around treatment stage, appliance type, expected pain timing, and chairside follow-up decisions. It is not intended to replace existing evidence-based interventions or function as a validated prediction rule.

This article is a narrative review rather than a systematic review or meta-analysis. The emphasis is on clinical interpretation and integration of heterogeneous evidence, not on pooled effect estimation. Accordingly, the review uses PRISMA-style transparency where feasible, but the proposed framework should be interpreted as a conceptual synthesis requiring prospective validation.

## Review

Methods

A narrative literature search was conducted to identify original studies and review articles relevant to orthodontic pain, discomfort, pain perception, patient-reported pain sensitivity, and individualized pain management. PubMed, Scopus, Web of Science, and Google Scholar were searched for articles published between 2014 and 2026, with older landmark studies included when they provided important background information on mechanisms, timing, assessment, or treatment of orthodontic pain. The last search was performed in May 2026.

A PICO-like framework was used to define the scope: the population was orthodontic patients; the exposure or clinical context included fixed appliances, clear aligners, separators, archwire insertion, appliance activation, elastics, debracketing, and adjunctive pain-control methods; comparators included different appliance types, intervention groups, placebo or no-intervention groups, and different treatment stages when available; and outcomes included pain intensity, pain timing, functional burden, OHRQoL, analgesic use, anxiety, satisfaction, adherence, and treatment-related daily life limitations.

The following keywords were used alone and in combination: orthodontic pain, orthodontic discomfort, pain perception, pain threshold, pain tolerance, patient-reported pain sensitivity, fixed orthodontic appliances, clear aligners, separator pain, bonding pain, debonding pain, elastics, low-level laser therapy (LLLT), photobiomodulation, transcutaneous electrical nerve stimulation (TENS), analgesics, nonsteroidal anti-inflammatory drugs (NSAIDs), acetaminophen, anxiety, anticipation of pain, catastrophizing, personality traits, patient education, digital follow-up, and personalized pain management. A representative PubMed search string is provided in Appendix A.

**Figure 1:**
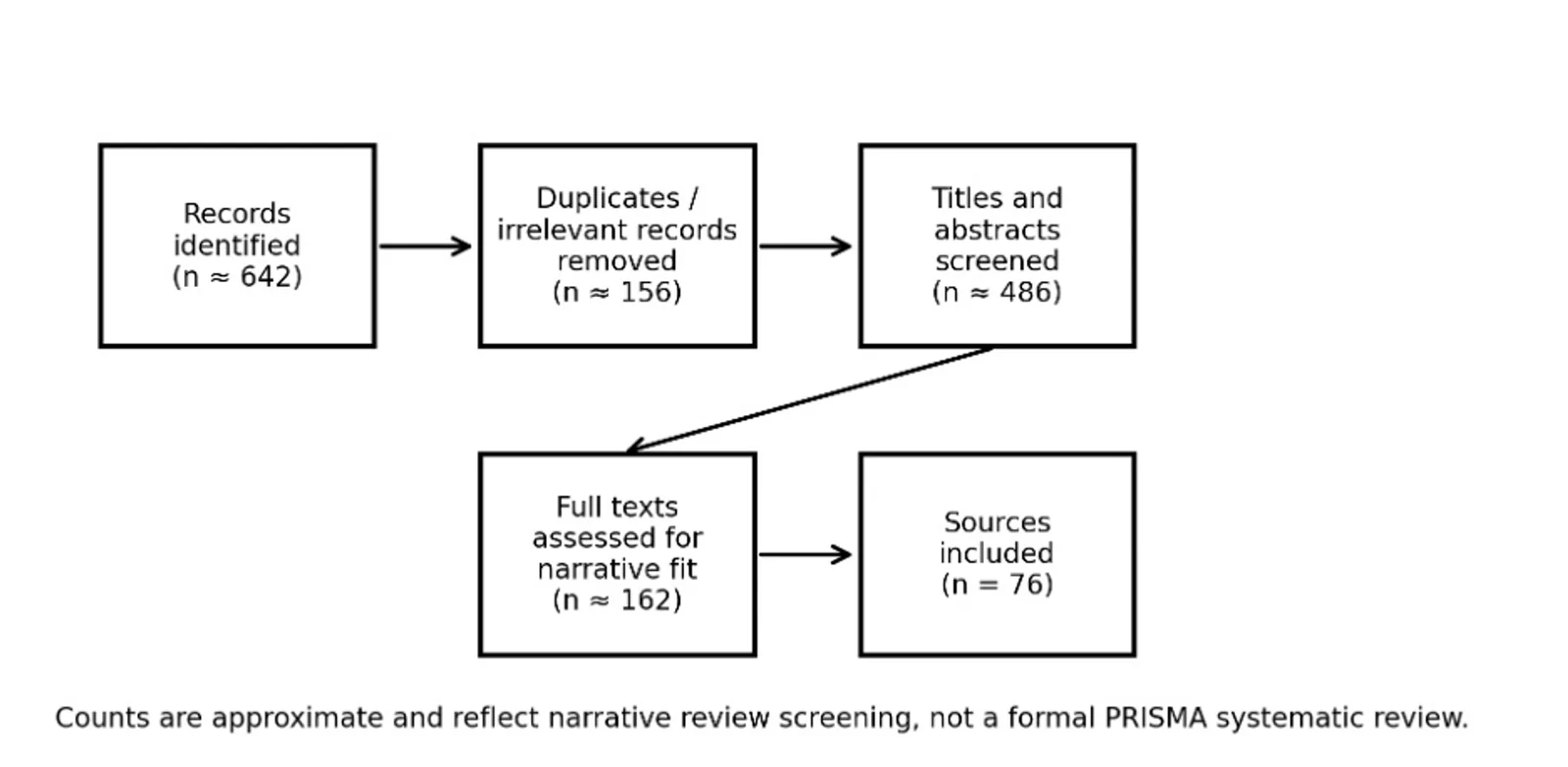
Narrative review literature identification and selection flow

The initial searches and reference-list screening identified approximately 642 records. After removal of duplicates and clearly irrelevant records, approximately 486 titles and abstracts were screened for relevance to orthodontic pain, pain assessment, pain determinants, or pain management. Approximately 162 full-text articles were assessed for narrative relevance, and 76 sources were incorporated into the revised synthesis. These counts are approximate because the review was not prospectively designed as a systematic review. The selection process is summarized in Figure 1.

Priority was given to randomized controlled trials, observational clinical studies, prospective studies, experimental studies, systematic reviews, meta-analyses, and original patient-reported outcome studies published in English. Studies were excluded when they were not related to orthodontic patients or orthodontic procedures, did not address pain or pain-related patient-centered outcomes, were not available in English, or were not relevant to the clinical scope of this review. Manual screening of reference lists was used to identify additional foundational studies.

Because this article was designed as a narrative review, study selection was based on clinical relevance and conceptual contribution rather than on a formal systematic screening process. No formal risk-of-bias assessment, meta-analysis, or quantitative synthesis was performed. Therefore, intervention-related statements are interpreted cautiously and are presented as clinical appraisals rather than pooled estimates of effect.

Assessment of orthodontic pain

Visual Analogue Scale (VAS)

Because orthodontic pain is subjective, its assessment mainly depends on patient-reported scales [20,21]. The VAS is one of the most commonly used tools in orthodontic pain studies. It usually consists of a 10-cm or 100-mm horizontal line with no pain at one end and worst pain or unbearable pain at the other end. The patient marks the point that best represents perceived pain intensity, and the distance from the zero point provides a numerical pain score [22-24].

The VAS is widely used because it is simple, easy to understand, sensitive to changes in pain intensity, and suitable for repeated measures pain assessment during orthodontic treatment [20].

Numerical Rating Scale (NRS), Verbal Descriptor Scale (VDS), and Orthodontic-Specific Instruments

The NRS allows patients to express pain intensity using numbers, usually from 0, indicating no pain, to 10 or 100, indicating the worst possible pain. The VDS evaluates pain intensity using descriptive categories such as no pain, mild pain, moderate pain, severe pain, or unbearable pain. Numerical scales are practical and easy to administer, whereas verbal scales may be helpful in routine practice but can be less sensitive for detecting small changes in pain intensity [21,22]. Orthodontic-specific instruments such as the Multidimensional Pain Inventory adapted for orthodontic patients may capture broader behavioral and psychosocial aspects of pain than a single intensity score [25].

Timing of Pain Assessment

The timing of pain assessment is important because orthodontic pain changes rapidly after force application. Pain usually begins within the first few hours, reaches its highest level around 24-48 hours, and then gradually decreases during the following days [4-7]. Therefore, repeated measurements are more informative than a single pain score. Time points such as zero, two, four, six, 12, 24, and 48 hours can provide a detailed short-term pain profile after archwire placement, separator use, appliance activation, or adjunctive intervention.

Need for Multidimensional Assessment

Pain intensity alone may not fully reflect the patient’s orthodontic pain experience. For risk-based pain management, additional patient-centered outcomes should be considered, including anxiety level, anticipation of pain, catastrophizing, analgesic use, sleep disturbance, chewing difficulty, dietary changes, daily life limitations, oral hygiene problems, satisfaction, and treatment adherence [25-28]. A multidimensional assessment is clinically important because two patients with similar pain scores may differ in psychological distress, functional burden, analgesic need, and treatment tolerance.

Biological determinants of orthodontic pain

Inflammatory Basis and Periodontal Ligament Remodeling

Orthodontic pain mainly results from the biological response of periodontal tissues to mechanical force. When force is applied through separators, brackets, archwires, aligners, elastics, or orthopedic appliances, compression and tension areas develop in the periodontal ligament. This process induces vascular changes, temporary ischemia, cellular signaling, and remodeling of periodontal ligament and alveolar bone tissues [2,9,29]. The delayed onset and peak of pain during the first 24-48 hours are compatible with an inflammatory response pattern rather than only immediate mechanical pressure [4,5,7,30].

Inflammatory mediators such as prostaglandins, bradykinin, histamine, serotonin, substance P, and cytokines contribute to nociceptor sensitization during orthodontic tooth movement [2,9]. IL-1 beta is particularly relevant because it is involved in inflammation and bone remodeling. Studies evaluating different magnitudes of continuous orthodontic force reported that stronger forces may increase IL-1 beta levels and pain intensity without necessarily producing more efficient tooth movement [31]. More recent work suggests that inflammatory cytokine profiles may interact with anxiety and catastrophizing, supporting a biopsychosocial interpretation of orthodontic pain [11].

Pain Threshold, Pain Tolerance, and Patient-Reported Pain Sensitivity

Inflammation alone cannot fully explain orthodontic pain. Pain threshold is the lowest stimulus intensity perceived as painful, whereas pain tolerance is the maximum pain level a patient can endure. In this review, the term “patient-reported pain sensitivity” is used as a broad clinical descriptor for a patient’s subjective tendency to notice, rate, and be functionally affected by pain. It does not imply a validated biological threshold unless formal sensory testing is performed. These constructs are clinically important because two patients receiving similar orthodontic forces may report very different pain levels [20,25]. Self-reported tools such as the VAS, NRS, VDS, and orthodontic pain questionnaires are therefore essential in research and practice [21,22].

Patient-reported pain sensitivity should also be interpreted in a broader patient-centered context. Orthodontic pain can affect eating, sleep, daily routine, emotional response, and satisfaction, and some patients may experience a mismatch between clinician expectations and patient-perceived functional burden [26,27,32].

Genetic Susceptibility

Genetic factors may partly influence individual pain responses. Genetic polymorphisms have been associated with pain modulation and differences in orofacial pain sensitivity, although the evidence is heterogeneous [33]. The catechol-O-methyltransferase gene has been associated with neural pain processing in broader pain research [34]. Orthodontic-specific data remain limited, and available clinical evidence suggests that genetic markers currently have weaker predictive value than procedure-related and psychological factors [10].

At present, available orthodontic evidence does not support routine clinical use of genetic markers to predict orthodontic pain. In one clinical study, bonding procedures and pain catastrophizing were stronger predictors of orthodontic pain than age, sex, or the investigated genetic markers [10]. Genetic susceptibility should therefore be considered a promising research area rather than a practical chairside tool at present.

Age, Sex, and Demographic Modifiers

Age and sex have been studied as possible modifiers of orthodontic pain, but findings are inconsistent. Some studies suggest that adolescent girls may report higher pain levels or different pain trajectories, whereas other studies have not found a stable relationship between pain intensity and age or sex [5,10,35]. These variables should therefore be interpreted together with psychosocial traits, pain history, pain expectation, procedure type, appliance design, and individual pain sensitivity rather than as independent predictors.

In clinical practice, demographic features should be evaluated together with anxiety, catastrophizing, previous dental experiences, and the planned procedure. A teenager with low anxiety and positive dental experiences may require only basic counseling, whereas an adult with high anxiety, previous painful dental treatment, and complex mechanics may require closer monitoring despite having no obvious demographic risk factor.

Psychological determinants of orthodontic pain

Anxiety and Anticipation of Pain

Psychological factors are central to the orthodontic pain experience. Dental anxiety may increase attention to discomfort, reduce tolerance, and intensify the subjective meaning of routine treatment-related pain [11,26,36]. Patients who anticipate severe pain may become hypervigilant during and after appliance placement, and this expectation may amplify the perceived intensity of otherwise transient discomfort [1,11]. For this reason, anxiety and anticipation of pain should be assessed before procedures known to produce discomfort, particularly separator placement, bonding, initial archwire insertion, activation appointments, elastic wear, and debracketing.

Prospective evidence indicates that anxiety can influence pain perception and daily routine after fixed appliance installation, while follow-up communication such as text messages may reduce pain levels and negative effects on daily activities [26]. This supports a clinically practical point: pain management may begin before the inflammatory peak by reducing uncertainty, setting realistic expectations, and offering timely follow-up during the first 24-72 hours.

Previous Dental Experiences, Decision-Making, and Concordance

Previous unpleasant or painful dental experiences can shape how patients interpret orthodontic stimuli. A patient who remembers earlier difficult dental treatment may anticipate similar pain during orthodontic procedures, even when the planned intervention is routine [3,36]. This anticipatory response may increase fear and perceived pain intensity.

Shared decision-making and patient concordance are therefore relevant to pain management, not only to treatment acceptance. Clear communication and perceived support may influence patient experience and satisfaction during orthodontic treatment [37]. Asking about previous dental pain, treatment anxiety, and personal concerns before high-risk procedures is a practical way to identify patients who may require more detailed reassurance, written information, or closer follow-up.

Pain Catastrophizing, Personality Traits, and Emotional Response

Pain catastrophizing refers to an exaggerated negative cognitive and emotional response to actual or expected pain. Patients with high catastrophizing tendencies may focus excessively on discomfort, feel less able to cope with pain, and interpret normal orthodontic soreness as threatening. Original clinical evidence suggests that catastrophizing and personality-related variables may be relevant to pain perception during orthodontic treatment, although results differ between studies and treatment phases [10-13].

Because catastrophizing may interact with inflammatory markers, orthodontic pain should be viewed as a biopsychosocial experience rather than as a simple tissue reaction [11]. Chairside management does not require formal psychological diagnosis; rather, it requires recognition of high-risk features such as repeated worry, very negative expectations, low confidence in coping, avoidance of eating or brushing, and repeated reassurance-seeking after routine procedures.

Communication, Reassurance, and Behavioral Support

Clear communication is a low-risk and clinically important part of risk-based pain management. Patients should be informed that orthodontic pain is usually temporary, commonly peaks within the first 24-48 hours, and gradually resolves. Explaining this expected course may reduce uncertainty and help patients distinguish normal treatment discomfort from warning signs requiring clinical attention [2,7,30]. Recent randomized trials suggest that written or multimedia information may not always reduce pain scores directly; however, structured education can improve understanding, reduce uncertainty, and support more effective communication at the start of treatment [38,39].

In this review, communication is considered a first-line behavioral strategy that can be delivered chairside by the orthodontic team. It is distinct from formal cognitive behavioral therapy or structured psychological interventions, which require specific training and are discussed as adjuncts for selected patients. Behavioral and attitude-modification interventions, including structured telephone calls, cognitive behavioral approaches, reassurance, relaxation, and other psychological strategies, have been discussed as potential adjuncts for reducing orofacial pain in orthodontic patients [40,41]. Patient satisfaction is also influenced by communication quality, perceived support, psychosocial expectations, and social experiences during treatment [37]. Therefore, communication should be considered a therapeutic component of pain management rather than a simple administrative explanation.

Clinical determinants of orthodontic pain

Bonding and Initial Archwire Placement

Bonding and initial archwire placement represent clinically important moments because they introduce a new orthodontic stimulus and often coincide with the patient’s first direct experience of fixed appliance discomfort. Pain generally begins within the first few hours after appliance placement, reaches its highest level around 24 hours or within the first 24-48 hours, and gradually decreases over the following days [4-7]. Questionnaire-based and observational studies show that the first week may be associated with chewing difficulty, dietary changes, daily discomfort, analgesic use, and reduced routine function [27,32,42,43].

Clinically, the initial archwire period should be treated as a high-response phase, particularly in anxious, pain-sensitive, or first-time orthodontic patients. Pretreatment counseling should include realistic information about timing, expected severity, soft diet advice, hygiene maintenance, appropriate analgesic use, and when to contact the clinician if pain is severe, asymmetric, associated with appliance injury, or prolonged beyond the expected course.

Separators

Separator placement is another procedure commonly associated with pain and chewing discomfort. Pain after separation usually increases during the first hours, peaks around 24 hours, and decreases over the following days [44,45]. Separator-related pain has been evaluated in trials of preoperative analgesics, topical or localized anti-inflammatory approaches, chewing gum, and photobiomodulation [16,46-48]. Because separators are frequently used before molar banding, this phase should be considered a procedure-specific risk period, especially for anxious or pain-sensitive patients.

Elastics

Intermaxillary elastics may also cause clinically relevant pain and may reduce patient cooperation. Tuncer et al. reported that pain related to intermaxillary elastics may begin within the first hours, increase between six hours and the first night, and then decrease by the end of the first week [14]. Since elastics require active patient cooperation, pain control, clear instructions, and early follow-up are important for maintaining adherence.

Debracketing and End-of-Treatment Discomfort

Pain and discomfort are not limited to the start of treatment. Debracketing can cause pressure, vibration-related discomfort, enamel-surface concerns, and anxiety, especially in patients who are apprehensive about instrument use or sudden forces. Clinical studies on debonding procedures suggest that pain can vary according to bracket type, debonding method, tooth location, and patient perception [15]. A recent randomized controlled trial reported that biting on a cotton roll during metal bracket debonding with Weingart pliers reduced pain scores, particularly in anterior regions, suggesting a simple chairside stabilizing method that may be useful during debonding [49]. Including debonding in pain counseling may help patients understand that transient discomfort can occur at several treatment stages, not only after appliance activation.

Appliance Type

Appliance type may influence both the intensity and quality of orthodontic pain. Fixed labial appliances are generally associated with discomfort after bonding, archwire placement, and activation, as well as mucosal irritation and food-related limitations. Lingual appliances may create a different burden because tongue irritation, speech interference, and hygiene challenges can add to pain-related discomfort. Clear aligners may produce intermittent pressure during aligner changes and may cause soft-tissue irritation from aligner edges or attachments.

Comparative evidence suggests that clear aligner patients may report better OHRQoL and lower pain at some early stages, although findings are not uniform across all phases, populations, and outcome measures [17-19]. Removability may reduce continuous pain for some aligner patients, but it may also introduce behavioral variability because patients can remove the appliance during discomfort. Fixed appliances may produce more mucosal irritation and food-related limitations, whereas aligners may shift the burden toward compliance, hygiene, and repeated activation cycles. Appliance type should therefore be considered when discussing expected pain patterns and when planning patient-specific pain management [30].

Force Magnitude and Treatment Mechanics

The magnitude, duration, and distribution of orthodontic force are important clinical determinants of pain. Heavy or poorly controlled forces may create greater periodontal compression, ischemia, and inflammatory mediator release, whereas light and controlled forces are biologically more favorable [2,29,31]. Treatment mechanics also matter. Continuous forces from fixed appliances may produce a different pain profile than intermittent forces from removable appliances. Orthopedic forces, such as maxillary expansion or extraoral traction, may cause pressure and discomfort in broader craniofacial regions rather than only tooth-related pain.

Overall, orthodontic pain should not be managed with the same approach at every stage of treatment. The most painful periods, such as separator placement, bonding and initial archwire insertion, activation visits, elastic use, and appliance removal, should be anticipated in advance. A risk-based strategy based on procedure type, force level, appliance design, and patient-reported pain sensitivity may improve comfort, cooperation, and treatment adherence. The main determinants of orthodontic pain are summarized in Table 1, with supporting references cited directly in the table.

**Table 1:** Biological, psychological, and clinical determinants of orthodontic pain OHRQoL, oral health-related quality of life

Determinant	Response or predictor	Clinical relevance	Implication for risk-based management
Inflammatory periodontal response [2,9,29,31]	Compression/tension zones, vascular change, mediator release, cytokine activity, and nociceptor sensitization	Explains delayed onset and 24- to 48-hour peak pain	Use controlled forces and explain the expected pain course
Pain threshold, tolerance, and patient-reported pain sensitivity [20-22,25]	Variation in nociceptive sensitivity, coping capacity, and reporting style	Similar forces may produce different pain experiences	Identify pain-sensitive patients and consider repeated measures pain assessment or diaries
Genetic susceptibility [10,33]	Pain-related polymorphisms may influence pain modulation	Evidence remains insufficient for routine clinical prediction	Research area; routine genetics-based chairside prediction is not recommended
Demographic and psychosocial traits [5,10-13,35]	Age, sex, anxiety, anticipation of pain, and catastrophizing may modify pain reporting and coping	Findings vary, but psychological features may amplify pain and functional limitation	Interpret demographics together with anxiety, pain history, expectation, and procedure type
Clinical procedure type [7,14,15,45]	Bonding, archwire insertion, separators, elastics, and debracketing differ in pain profile	Some stages affect chewing and cooperation more strongly	Prepare patients before high-risk appointments
Appliance type [17-19]	Different force delivery, mucosal irritation, removability, and hygiene demands	Pain and OHRQoL may differ between fixed appliances, lingual appliances, and aligners	Tailor counseling according to appliance and treatment stage
Force magnitude and mechanics [2,30,31]	Heavy or continuous forces may increase periodontal compression and inflammatory response	May increase pain without improving efficiency	Prefer controlled mechanics, especially in patients with higher pain risk

Current pain management strategies in orthodontics

Orthodontic pain can be managed with pharmacological and nonpharmacological approaches. In a risk-based pain management model, the question is not only which method has the largest average effect, but which approach is most suitable for a specific patient, procedure, clinical context, and contraindication profile. The main options are summarized in Table 2, with an explicit appraisal of the evidence base to distinguish relatively well-supported approaches from more emerging or heterogeneous methods.

**Table 2:** Evidence appraisal and clinical interpretation of pharmacological and nonpharmacological options for orthodontic pain control LLLT, low-level laser therapy; NSAIDs, nonsteroidal anti-inflammatory drugs; TENS, transcutaneous electrical nerve stimulation

Method	Evidence appraisal/clinical interpretation	Orthodontic application	Key cautions	Suitable use
Acetaminophen [46,50,51]	Relatively well-supported for short-term mild-to-moderate orthodontic pain when used appropriately	Procedure-related pain after separator placement, bonding, or archwire activation; generally limited to the first 24-72 hours as needed	Use age- and weight-appropriate dosing according to local guidance and product labeling; avoid exceeding maximum daily dose; consider liver disease, alcohol use, and drug interactions; consult a physician when uncertain	Low- to moderate-risk patients needing short-term analgesia
NSAIDs [46,50,52]	Effective for short-term pain relief, but routine or prolonged use should be avoided because tooth movement depends partly on prostaglandin-mediated remodeling	Selected moderate-to-severe pain after separators or archwire activation when medically appropriate	Use the lowest effective dose for the shortest duration, typically limited to early peak pain; consider gastrointestinal, renal, cardiovascular, asthma, anticoagulant, pregnancy, allergy, and intolerance risks; physician consultation may be required	Patients with higher pain intensity and no contraindications
Topical analgesics or patches [47]	Emerging localized option; evidence and protocols vary	Localized separator- or appliance-related discomfort	Avoid broad generalization; protocols, formulation, and mucosal tolerance vary	Selected localized discomfort when systemic medication is undesirable
LLLT/photobiomodulation [16,48,53-56]	Evidence is promising but heterogeneous; recent systematic and trial evidence suggests benefit in selected settings, especially early high-pain stages, but not uniform efficacy throughout treatment	Separator placement, initial archwire placement, post-adjustment pain, canine retraction, and selected activation appointments	Results depend on wavelength, dose, energy density, application timing, number of sessions, and appliance stage	Selected patients preferring nonpharmacological support or those with contraindications to medication
TENS [57-60]	Emerging adjunct with limited orthodontic-specific evidence	Potential adjunct during early fixed-appliance pain or in drug-avoidant patients when device access and expertise are available	Device settings, electrode placement, frequency, and duration vary; avoid overstatement of efficacy	Selected high-risk or drug-avoidant patients
Vibration and chewing exercises [55,61,62]	Moderate but variable evidence; simple physical methods may help some patients by sensory modulation and periodontal blood-flow effects	Chewing gum, bite wafers, or controlled chewing exercises after initial archwire placement in cooperative fixed-appliance patients	Consider bracket failure risk, chewing restrictions, patient compliance, and appliance type	Patients seeking simple adjunctive nonpharmacological support
Low-intensity pulsed ultrasound [63,64]	Emerging and mainly investigational for pain; more evidence relates to tissue remodeling and tooth movement than patient-centered pain relief	Research settings or selected clinical contexts with appropriate equipment	Do not equate tooth-movement or remodeling effects with proven pain relief	Selected patients or research settings
Behavioral support, education, and digital follow-up [26,38-41,65]	Low-risk and clinically practical; effects on pain intensity vary, but communication can reduce uncertainty and improve coping	Preprocedure education, written instructions, 24- to 72-hour follow-up, digital pain diary, or text-message support	Requires patient engagement and digital access; formal psychological interventions require appropriate training	All patients, especially anxious or high-risk patients

Pharmacological Methods

Analgesics are commonly used because they are easy to prescribe and provide relatively rapid relief. Acetaminophen is often preferred for mild to moderate orthodontic pain because it has limited peripheral prostaglandin inhibition and is generally considered appropriate for short-term dental pain management when no contraindication exists [50,51]. In orthodontic practice, its use should be limited to short-term, as-needed pain control, usually during the early peak period after separators, bonding, or archwire activation. Dosing should follow age- and weight-appropriate local prescribing guidance and product labeling, and patients should be advised not to exceed the maximum daily dose. Patients with liver disease, heavy alcohol use, complex medical histories, pregnancy, or concurrent medications should be advised to consult a physician or pharmacist before use.

NSAIDs, such as ibuprofen and naproxen, may provide effective short-term pain relief after separator placement or archwire activation [46,50]. However, because orthodontic tooth movement depends partly on inflammation and prostaglandin-mediated bone remodeling, unnecessary or prolonged NSAID use should be avoided [2,50]. NSAID recommendations should therefore emphasize the lowest effective dose for the shortest period, generally limited to the first 24-72 hours when clinically needed, and should consider contraindications such as gastrointestinal disease, renal disease, cardiovascular risk, anticoagulant use, asthma sensitivity, pregnancy, allergy, and drug intolerance. Prescription decisions should be based on medical history, contraindications, and clinician judgment rather than preference alone.

Recent analgesic-focused evidence supports the effectiveness of analgesics for short-term orthodontic pain, but it also reinforces the need for careful patient selection, dosing, timing, and avoidance of routine prolonged use [50]. Topical or localized analgesic strategies, including naproxen patches, have been investigated as alternatives to systemic medication in selected settings [47]. Pharmacological pain control should therefore be short-term, procedure-specific, and based on individual pain severity, medical history, contraindications, allergies or intolerance, and consultation with a physician when needed.

LLLT and Photobiomodulation

LLLT, also described as photobiomodulation, is one of the most frequently studied drug-free methods for orthodontic pain control. Proposed mechanisms include modulation of inflammatory mediator release, mitochondrial activity, peripheral nerve excitability, microcirculation, and nociceptor sensitization. Randomized clinical studies suggest that diode LLLT may reduce orthodontic pain in selected settings, but treatment protocols vary considerably between studies [53]. Clinical trials have assessed LLLT after post-adjustment pain, fixed appliance activation, canine retraction, separator placement, and initial archwire placement using different wavelengths, doses, irradiation schedules, and outcome time points [48,54,66-73]. This protocol heterogeneity helps explain why findings are not fully consistent.

Some studies reported reduced pain, whereas others showed limited or context-dependent effects. A recent systematic review on separation pain reported that LLLT was effective in reducing pain after separator placement, but the included trials differed in protocols and time points [16]. A recent randomized controlled trial comparing LLLT with paracetamol-caffeine across several stages of leveling and alignment found that LLLT reduced peak pain in selected early stages, particularly separation, but was not uniformly effective throughout the entire course of treatment [56]. Therefore, photobiomodulation may be considered selectively for patients preferring nonpharmacological support or when medication is unsuitable, but it should not be presented as a universally effective intervention.

TENS and Other Physical Interventions

TENS is a noninvasive method that may reduce pain through stimulation of large-diameter sensory fibers and modulation of nociceptive transmission at spinal and trigeminal levels, consistent with gate-control concepts of pain modulation [57,58]. In practical terms, nonpainful sensory input may inhibit or dampen transmission from smaller nociceptive fibers, reducing the perceived intensity of pain. Early orthodontic research evaluated the effect of TENS on pain associated with tooth movement [59]. More recent orthodontic reports describe TENS or related dental pain-erasing devices as potential adjuncts, but the evidence remains less standardized than that for analgesics or photobiomodulation [60,74].

Other nonpharmacological strategies include vibration, chewing exercises, chewing gum, cold application, behavioral support, patient education, low-intensity pulsed ultrasound, and digital follow-up. Physical interventions may work through several mechanisms, including competing sensory stimulation, improved periodontal blood flow, muscle relaxation, distraction, and modulation of local inflammatory responses. Original clinical studies suggest that physical interventions may have potential benefits, but the strength and consistency of evidence differ among methods and protocols [61,75]. Chewing gum has also been proposed as a low-cost option for reducing pain during fixed appliance treatment, although its use should be considered together with bracket failure risk and patient-specific factors [62].

Low-intensity pulsed ultrasound has been investigated in relation to bone remodeling and tooth movement, but these biological outcomes should not be directly equated with patient-centered pain relief [63,64]. Therefore, this method should be regarded as emerging for orthodontic pain management and more suitable for research contexts or carefully selected clinical settings than for routine pain-control recommendations.

Behavioral, Educational, and Digital Strategies

Behavioral and educational strategies should be incorporated into orthodontic pain management because pain is influenced by anxiety, expectation, coping style, and patient-clinician communication. Original clinical studies have evaluated cognitive behavioral strategies, structured follow-up, and reassurance as potentially useful adjuncts for reducing pain-related distress in orthodontic settings [40]. Broader pain literature also supports integrated psychological approaches as part of pain management, particularly when pain experience is shaped by cognitive and emotional factors [41].

Digital follow-up can personalize pain care by identifying patients whose pain is more severe or prolonged than expected. Text-message follow-up has been associated with reduced pain perception and reduced negative impact on routine in orthodontic patients [26]. Smartphone-based ecological momentary assessment can collect real-time pain scores in daily environments and may reduce recall bias compared with retrospective pain reporting [65]. These tools may be particularly useful during the first 48-72 hours after bonding, archwire insertion, separator placement, or aligner activation.

A conceptual risk-based management pathway for orthodontic pain

Because orthodontic pain is shaped by biological responses, psychological status, and treatment-related factors, a uniform pain management strategy is unlikely to be optimal for every patient. Current evidence suggests that pain intensity and treatment tolerance are influenced not only by the type of orthodontic procedure but also by anxiety, catastrophizing, previous dental experiences, pain threshold, appliance type, force magnitude, communication quality, and patient expectations [2,10,11,17,26,37]. The framework proposed in this review should be interpreted as a conceptual management pathway rather than a validated clinical prediction tool. It is intended to help clinicians structure pain risk assessment and tailor support according to patient needs.

The proposed pathway differs from a general biopsychosocial description in three ways. First, it translates biological, psychological, and clinical determinants into orthodontic decision points before specific procedures. Second, it links risk level to practical actions, including counseling intensity, analgesic planning, appliance- and procedure-specific advice, and follow-up frequency. Third, it emphasizes reassessment during the first 24-72 hours, when orthodontic pain commonly peaks. Thus, the framework should be viewed as a chairside conceptual guide for management planning, not as a validated scoring system.

**Figure 2:**
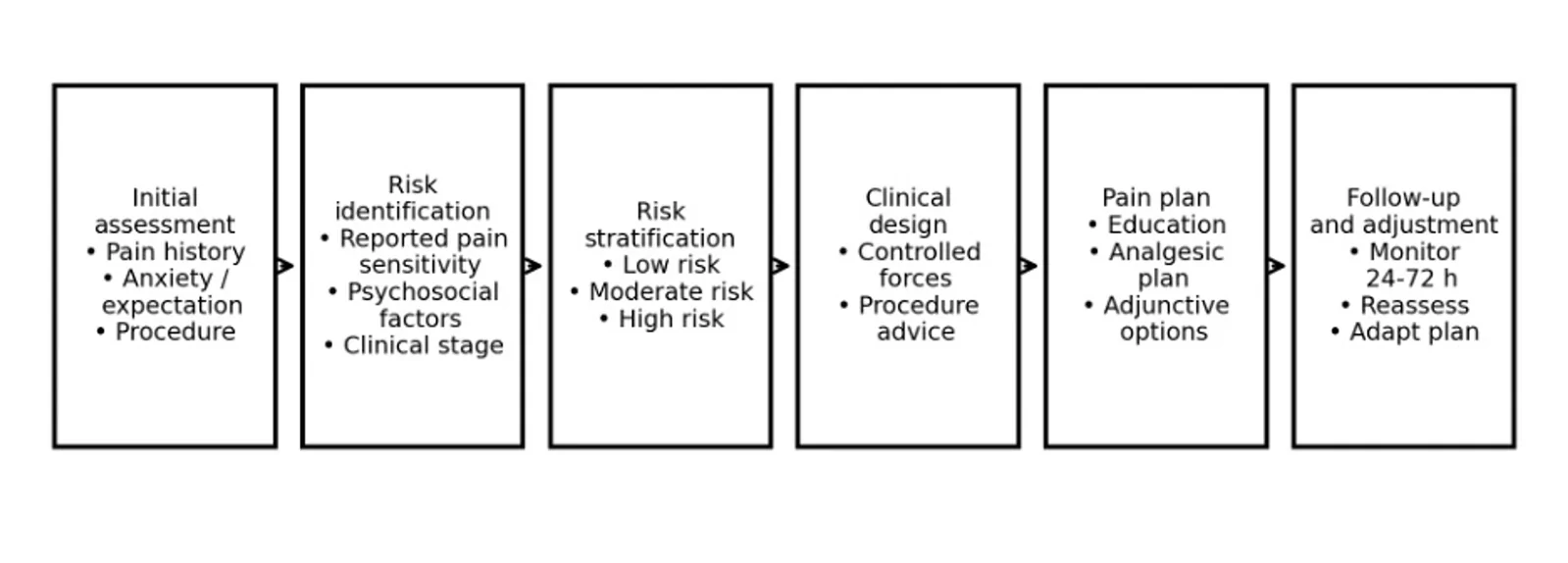
Proposed conceptual risk-based orthodontic pain management pathway

Biological determinants are not classified through genetic testing or laboratory thresholds in routine practice. Instead, risk identification relies on patient-reported pain history, usual pain tolerance, previous response to dental procedures, anxiety, anticipation of pain, procedure type, appliance design, and expected force level. The risk-based approach is summarized in Table 3 and Figure 2.

**Table 3:** Conceptual risk-based pain management pathway according to patient risk profile

Risk group	Patient-reported and clinical risk features	Recommended approach	Follow-up strategy
Low risk [4,7]	Low anxiety, good coping confidence, positive dental experience, mild crowding, and simple mechanics	Pretreatment explanation, soft diet advice, expected pain timing, reassurance, and short-term acetaminophen if needed and medically appropriate	Routine follow-up; advise contact if pain is severe or prolonged
Moderate risk [5,6,14,45]	Moderate anxiety, previous unpleasant dental experience, moderate crowding, initial archwire, separator, or elastic use	Detailed pain education, first 48-hour instructions, short-term analgesic plan when appropriate, and simple nonpharmacological support	Telephone or message follow-up during the first 24-48 hours after painful procedures
High risk [10-13]	High anxiety, low coping confidence, high pain anticipation, catastrophizing, previous traumatic dental experience, complex mechanics, or poor cooperation risk	Detailed counseling, expectation management, gradual/light mechanics where feasible, individualized analgesic plan, and selected adjunctive methods when appropriate	Frequent early follow-up, digital pain monitoring, reassessment after high-risk procedures, and behavioral support if needed

Low-Risk Patients

Low-risk patients generally present with low anxiety, good coping confidence, positive previous dental experiences, mild crowding, and relatively simple treatment mechanics. In these patients, orthodontic pain is often self-limiting and follows the expected pattern of early onset, peak discomfort within the first 24-48 hours, and gradual reduction thereafter [4,7]. Basic supportive care is usually sufficient, including pretreatment education, explanation of the expected time course of pain, soft diet advice, reassurance, and short-term acetaminophen if needed and medically appropriate [50,51].

Moderate-Risk Patients

Moderate-risk patients may present with moderate anxiety, previous unpleasant dental experiences, moderate crowding, or treatment stages known to produce more discomfort, such as initial archwire placement, separator placement, or elastic use [6,14,45]. These patients may benefit from detailed pain education, a simple pain diary, first 48-hour instructions, short-term analgesic support when appropriate, and selected nonpharmacological measures such as controlled chewing exercises or cold application [50,61,62].

Patient education may be delivered verbally, in written form, through multimedia tools, or by text-message follow-up. Recent randomized trials suggest that additional written or multimedia information may not always directly reduce pain or anxiety scores, but structured education may improve patient understanding, reduce uncertainty, and support more efficient clinical communication [38,39]. Text-message follow-up may be particularly useful in patients who are anxious or concerned about the effect of pain on routine activities [26].

High-Risk Patients

High-risk patients may have high anxiety, low coping confidence, high anticipation of pain, pain catastrophizing, previous negative dental experiences, marked crowding, complex mechanics, or a known risk of poor cooperation [10-13]. Management should begin before the painful procedure. Detailed counseling, expectation management, repeated reassurance, written or multimedia information, and early follow-up during the first days after appliance placement may help reduce uncertainty and improve coping [26,38,39].

When possible, lighter and more gradual mechanics may be preferred because higher force levels and more demanding clinical procedures may increase pain intensity [2,31]. Pain follow-up may need to be more frequent, and an analgesic plan can be discussed in advance. Adjunctive methods such as photobiomodulation, TENS, or selected physical interventions may be considered selectively, but they should not be applied uniformly to all patients because results vary across studies and protocols [53,60,61].

Clinical implications, future directions, and limitations

The main clinical implication of this review is that orthodontists should evaluate not only the malocclusion and planned mechanics but also the patient’s pain risk before treatment begins. A brief pain risk assessment may include questions about previous painful dental experiences, anxiety about orthodontic treatment, expected pain severity, usual pain tolerance, catastrophizing-type concerns, and fears about eating, sleeping, brushing, or daily activities after appliance placement [10,11,26,37]. This assessment can be incorporated into routine history taking and does not require complex equipment.

Procedure-specific counseling is especially important before separator placement, bonding, initial archwire insertion, elastic use, and debracketing because these stages are commonly associated with discomfort and functional limitation [7,14,15,45]. Original randomized and observational studies also support a risk-based approach to orthodontic pain control. Pharmacological interventions may provide short-term pain reduction, whereas structured communication, vibration therapy, LLLT, and other nonpharmacological strategies may have moderate or context-dependent benefits [53,61,75]. These findings suggest that nonpharmacological methods may be suitable for patients with mild to moderate pain or those for whom medication is unsuitable, while pharmacological support may be more appropriate for patients with severe pain or higher pain risk when no contraindication exists.

In daily practice, risk-based pain management may improve comfort, cooperation, and treatment adherence. It may also help reduce unnecessary analgesic use and prevent pain-related dissatisfaction. By anticipating pain risk before treatment and adapting communication, analgesic advice, appliance mechanics, and follow-up intensity to individual needs, orthodontists can provide more patient-centered care.

Future studies should move from a one-size-fits-all model toward prediction-informed and patient-centered orthodontic pain management. Patients should be evaluated according to baseline pain threshold, pain tolerance, dental anxiety, pain expectation, previous pain experience, pain catastrophizing, appliance type, procedure type, and treatment mechanics. Including these variables may help explain why patients exposed to similar orthodontic forces report different pain intensities [10-13]. Genetic markers remain a research interest, but their clinical value should be tested prospectively alongside psychological and clinical predictors [33].

Pain assessment should also be standardized and broadened. Many studies still report pain at isolated time points, although orthodontic pain changes rapidly during the first hours and days after force application. Future trials should assess the early pain profile with repeated measurements, particularly during the first 0-48 hours, when pain usually begins, increases, and reaches its peak [4-7]. Outcomes such as VAS area under the curve, analgesic use, chewing difficulty, sleep disturbance, dietary changes, adherence, oral hygiene difficulty, daily life limitation, and patient satisfaction may provide a more clinically meaningful assessment than a single pain score [25,27,28,37].

Digital pain monitoring may improve the accuracy of future research. Smartphone-based ecological momentary assessment can collect real-time pain scores in patients’ daily environments and may reduce recall bias compared with paper-based pain diaries [65]. Such tools could be especially useful for monitoring pain during the first 48-72 hours after bonding, archwire insertion, separator placement, aligner activation, or elastic use. Future trials should also test whether digital follow-up reduces unscheduled visits, analgesic overuse, dietary limitation, and anxiety-related escalation of pain.

Standardization is also needed for nonpharmacological methods. Photobiomodulation, TENS, vibration, chewing exercises, low-intensity pulsed ultrasound, and other physical interventions remain promising but inconsistent approaches, partly because studies differ in wavelength, dose, electrode placement, device settings, number of sessions, timing, and patient selection [53,60,61,64,75]. Future trials should use reproducible protocols and should identify which patient groups are most likely to benefit from each intervention.

Comparative studies should also evaluate risk-based pain management in fixed appliance, lingual appliance, and clear aligner patients. Appliance type may influence pain intensity, functional limitation, anxiety, adherence, and OHRQoL [17-19]. This is particularly important because much of the available intervention evidence is derived from adolescent fixed-appliance populations, and the applicability of the proposed framework to adults, clear aligner patients, and patients with comorbidities such as chronic pain conditions or psychiatric diagnoses has not yet been tested.

This narrative review has several limitations. First, the search and study selection process was not designed as a systematic review, and no formal risk-of-bias assessment or quantitative synthesis was performed. Second, the included evidence is heterogeneous in terms of patient age, appliance type, orthodontic procedure, pain measurement timing, outcome measures, and intervention protocols. Third, some included sources are older or lower-level original studies and were used mainly to provide historical or contextual support rather than to support definitive clinical claims. Fourth, the proposed risk-based pathway is conceptual and has not yet been prospectively validated as a clinical prediction tool or clinical protocol. Therefore, the recommendations should be interpreted as practical guidance for clinical reasoning rather than as definitive evidence-based protocols.

## Conclusions

Orthodontic pain is a multidimensional experience shaped by biological tissue responses, psychological status, and clinical treatment-related factors. A standardized approach may be insufficient because patients differ in pain threshold, pain tolerance, anxiety, anticipation of pain, catastrophizing, previous dental experiences, appliance type, treatment stage, and functional burden. The expanded literature supports the need to anticipate pain risk before high-discomfort procedures and to tailor communication, analgesic advice, appliance mechanics, adjunctive strategies, and follow-up intensity according to individual risk. The proposed model should be understood as a conceptual management pathway for structuring clinical reasoning, not as a validated clinical prediction tool or a new definitive protocol. Future prospective studies are needed to validate practical risk assessment tools and individualized pain management strategies in orthodontic patients.

## Appendices

Appendix A

("orthodontic pain" OR "orthodontic discomfort" OR "pain perception" OR "pain threshold" OR "pain tolerance" OR "visual analogue scale" OR VAS OR "numerical rating scale" OR NRS OR "oral health-related quality of life" OR OHRQoL) AND ("fixed orthodontic appliance" OR "clear aligner" OR aligner OR separator OR archwire OR bonding OR debonding OR elastics OR "appliance activation") AND (analgesic OR acetaminophen OR NSAID OR ibuprofen OR naproxen OR "low-level laser therapy" OR photobiomodulation OR TENS OR vibration OR "chewing gum" OR education OR anxiety OR catastrophizing OR "digital follow-up")
